# A bacteriophage transcription regulator inhibits bacterial transcription initiation by σ-factor displacement

**DOI:** 10.1093/nar/gku080

**Published:** 2014-01-28

**Authors:** Bing Liu, Andrey Shadrin, Carol Sheppard, Vladimir Mekler, Yingqi Xu, Konstantin Severinov, Steve Matthews, Sivaramesh Wigneshweraraj

**Affiliations:** ^1^MRC Centre for Molecular Microbiology and Infection, Imperial College London, London SW7 2AZ, UK, ^2^Waksman Institute for Microbiology and Department of Molecular Biology and Biochemistry, Rutgers, The State University of New Jersey, Piscataway, NJ USA and ^3^St. Petersburg State Polytechnical University, St. Petersburg, Russia

## Abstract

Bacteriophages (phages) appropriate essential processes of bacterial hosts to benefit their own development. The multisubunit bacterial RNA polymerase (RNAp) enzyme, which catalyses DNA transcription, is targeted by phage-encoded transcription regulators that selectively modulate its activity. Here, we describe the structural and mechanistic basis for the inhibition of bacterial RNAp by the transcription regulator P7 encoded by *Xanthomonas oryzae* phage Xp10. We reveal that P7 uses a two-step mechanism to simultaneously interact with the catalytic β and β’ subunits of the bacterial RNAp and inhibits transcription initiation by inducing the displacement of the σ^70^-factor on initial engagement of RNAp with promoter DNA. The new mode of interaction with and inhibition mechanism of bacterial RNAp by P7 underscore the remarkable variety of mechanisms evolved by phages to interfere with host transcription.

## INTRODUCTION

Bacteriophages (phages) use an impressive array of mechanisms to inactivate or repurpose bacterial processes for their own developmental needs (1,2). Many phage genomes encode small proteins that specifically affect the multisubunit RNA polymerase (RNAp) of the bacterial host, inhibiting bacterial DNA transcription while promoting regulated phage DNA transcription (1,2). Illuminating the functional mechanisms of phage-encoded RNAp inhibitors at the molecular and structural level uncovers new ways by which bacterial transcription is thwarted by non-bacterial regulators of bacterial transcription.

Bacterial transcription begins with the association of a σ-factor with the catalytic ‘core’ of the RNAp (subunit composition α_2_ββ’ω; E) resulting in the formation of the RNAp holoenzyme (Eσ). The σ-factor confers promoter specificity on the RNAp, and in the case of the primary σ-factor, called σ^70^ in *Escherichia coli* (*Ec*) and related bacteria, allows the specific recognition of the consensus sequence elements located around positions −35 and −10 (with respect to the transcription start site at +1) of bacterial promoters in front of essential genes. Primary σ factors have four evolutionarily conserved regions (1–4), with regions 2 and 4 being the major determinants of recognition of the −10 and −35 promoter elements, respectively (3). On its own, σ^70^ of *Ec*, the best-studied protein of its class, cannot recognize promoters. On binding to the core RNAp, σ^70^ undergoes large conformational changes (4). In particular, σ^70^ region 4 interacts with the structurally conserved and flexible ‘flap’ domain of the β subunit (specifically with the β flap domain helix), and this interaction leads to physical movement that positions σ^70^ region 4 such that simultaneous interactions with consensus −10 and −35 promoter elements become possible (5). The initial engagement of Eσ^70^ with the promoter yields a closed promoter complex (RPc), which isomerizes into the transcription-initiation competent open promoter complex (RPo) [reviewed in (6)].

**Figure 1.**
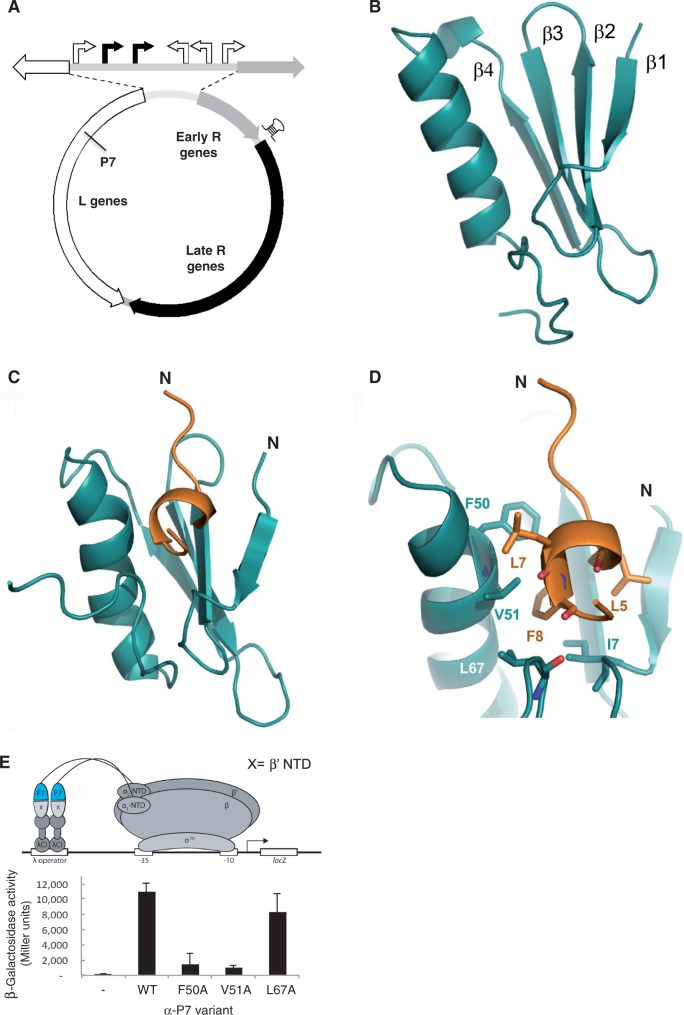
The solution structures of P7 and P7-β’ NTD complex. (**A**) The groups of Xp10 genes that belong to the different temporal classes are indicated on the Xp10 genome (shown in circular organization). The host (white arrows) and phage (black arrows) RNAp dependent promoters in the intergenic region separating the L genes and early R genes are shown at the top. In the L genes, the approximate location of the gene-encoded P7 is indicated and the putative transcription terminators that separate early and late R genes are indicated by a hairpin (see text for details). This figure has been adapted from Djordjevic *et al.* (12). (**B**) Cartoon representation of the solution structure of apo P7 showing the juxtaposition of the α helix and the β1 -β2-β3-β4 sheets. (**C**) Solution structure of P7 in complex with the first 10 aa residues of the β’ subunit from *Xo* RNAp (β’ NTD). P7 is shown in cyan and β’ NTD in orange. The position of N-termini for each chain is indicated. (**D**) Zoomed cartoon representation of the P7-β’ NTD complex showing residues located in the P7-β’ NTD binding site. Key interacting residues are labelled. (**E**) BTH interaction assay used to detect protein–protein interaction between β’ NTD and mutants of P7. The diagram depicts how the interaction between β’ NTD, fused to the bacteriophage λ CI protein (λCI), and P7, fused to the α-NTD (α-P7), activates transcription of the *lacZ* gene. Results of the β-galactosidase assays expressed in Miller units are shown in the bar chart.

Phages have evolved mechanisms to exploit the σ^70^ region 4/β flap interaction to repurpose the bacterial RNAp for transcription of phage genes (2). Known phage-encoded transcription regulators specifically interfere with the σ^70^ region 4/β flap interaction through direct contact with σ^70^ region 4, β flap or both, thereby altering transcription initiation and/or post-initiation activities of the bacterial RNAp (2,7–10). Lytic bacteriophage Xp10, which infects the bacterial phytopathogen *Xanthomonas oryzae* (*Xo*), the causal agent of rice blight, relies on the host RNAp and a phage-encoded single-subunit RNAp for the temporal expression of its genome. The transcription programme of Xp10 is complex and the precise regulatory basis for the temporal transcriptional regulation of Xp10 genes is not fully understood. The transcription of Xp10 genes (called L and early R genes; Figure 1A) during the early stages of infection is driven by at least four *Xo* Eσ^70^ dependent promoters, whereas the transcription of Xp10 genes during the late stages of infection (called late R genes; Figure 1A) is largely driven by Xp10 RNAp (11,12). The presence of a transcription terminator sequence located between early R genes and late R genes (Figure 1A) prevents any unwanted transcription of late R genes during the early stages of infection (11,12). A 8 kDa Xp10 protein, called P7, which is an L gene product and a strong inhibitor of RPo formation by the *Xo* Eσ^70^, is believed to facilitate the switching between host and phage RNAp for the transcription of Xp10 genes (13). P7 can also bind to transcribing host RNAp and function as an anti-terminator and allows the host RNAp to bypass the transcription terminator located between the early R genes and late R genes to increase transcription of late R genes during the late stages of infection by transcribing host RNAp (i.e. RNAp molecules that have initiated transcription before the production of P7 or have escaped P7 inhibition) (11–13). The first 10 amino acid (aa) residues of the β’ subunit of *Xo* Eσ^70^ contain the major determinant for P7 binding (14). Previous studies have shown that P7 affects the obligatory change in the interdomain distance between σ^70^ regions 2 and 4 that occurs during RNAp holoenzyme formation (13). However, the precise mechanism by which P7 inhibits RPo formation by *Xo* Eσ^70^ remains unknown. Here, we describe the structural and molecular basis for the interaction and mode of transcription inhibition by P7. We show that P7 uses a two-step mechanism to simultaneously interact with the catalytic β’ and β subunits of the bacterial RNAp and cause the displacement of σ^70^ on engagement of the RNAp holoenzyme with promoter DNA. Thus, P7 represents a distinct type of phage-encoded bacterial transcription regulator that inhibits transcription initiation of bacterial RNAp by σ-factor displacement.

## MATERIALS AND METHODS

### Plasmids and proteins

Plasmids encoding P7 fused to αNTD (pBRα) and different *Xo* and *Ec* β and *Xo* β’ fragments (details below) fused to the λ CI protein (pACλCI) and mutant versions thereof were constructed using standard recombinant DNA methods. Plasmids encoding amino-terminal heart-muscle-kinase–tagged (for labeling with γ-^32^P) P7 and *Ec* σ^70^ were constructed by cloning the relevant DNA sequences into plasmid pET33b+ (Novagen). Proteins were ^32^P-labelled in reactions containing 50 μl of protein kinase A (PKA) reaction buffer, 500 ng of protein, 1 μl of γ-^32^P-ATP (250 Ci/mmol, Perkin Elmer) and 5 U of PKA made up to 100 μl with ddH_2_0. Reactions were incubated at 37°C and stopped by rapid freezing in small aliquots in liquid nitrogen. The aliquots were stored at −80°C and discarded after thawing. Wild-type and P7-sensitive core RNAp was purified from *Ec* 397 c cells containing either pRL663^WT^ or pRL663 [which encodes the P7-sensitive β’ subunit; (14)], respectively, according to published protocols (15). The *Ec* σ^70^ was purified essentially as previously described (16). Details of all the oligonucleotide primers used for cloning and mutagenesis and plasmids used in this work are provided in Supplementary Tables S1 and S2. For nuclear magnetic resonance (NMR) spectroscopy and structure calculation, full-length P7 was expressed in pET-46 with EK/LIC site (Novagen). β’ NTD (MKDLLNLFNQ), β flap tip (KVTPKGESQLTPEEKLLRAIFGEKASDVKDS) and a further β’ NTD (MKDLL*NL*FNQ) in which two L* were uniformly ^13^C/^15^N-labelled were chemically synthesized (Peptide Synthetics, UK). P7 samples for NMR analyses were produced with uniform ^13^C/^15^N labelling by expressing in minimal medium supplemented with ^15^NH_4_Cl and ^13^C_6_-Glucose and purified using Ni-NTA agarose and size exclusion chromatography. To prepare a saturated sample of P7 bound to the β’ NTD peptide, the peptide was added to a dilute sample of purified P7 and incubated at room temperature overnight. The sample was concentrated by ultrafiltration, and the complex was obtained by size exclusion chromatography using a Superdex 75 column (GE Life Sciences).

### NMR spectroscopy and structure calculation

The backbone assignments of P7 and P7-β’ NTD complex were both completed by using standard double- and triple-resonance assignment approach (17). The data were processed and analysed in NMRView and our in-house assignment routines (18). Hα and Hβ assignments of both projects were obtained using HBHA(CBCACO)NH. The side-chain assignments of the two proteins were completed using HCCH TOCSY and (H)CC(CO)NH TOCSY. Proton resonance assignment of β’ NTD was based on ^12^C/^14^N-edited 2D homonuclear TOCSY/NOESY experiments, and to aid unambiguous assignment a sample was prepared in which the β’ NTD peptide was chemically synthesized with two leucine residues that were uniformly ^13^C/^15^N labelled (MKDLL*NL*FNQ). The distance restraints were provided by 3D ^1^H-^15^N/^13^C NOESY NOESY-Heteronuclear Multiple Quantum Coherence (HSQC) experiments (mixing time, 100 ms at 800 MHz). Intermolecular NOEs provide the distance restraint for the complex and were acquired using filter ^13^C/^15^N NOESY experiment on hybrid-labelled sample. The ARIA protocol was used for completion of the NOE assignment and structure calculation (19). Dihedral angle restraints derived from TALOS were also incorporated in the calculation. The frequency window tolerances for assigning NOEs were ±0.04 ppm for direct proton dimensions and ±0.05 ppm for indirect proton dimensions, and ±0.5 ppm for nitrogen dimensions and ±1.1 ppm for carbon dimensions. The ARIA parameters p, Tv and Nv were set to default values. The 20 lowest energy structures had no NOE violations >0.5 Å and no dihedral angle violations >5°. Dihedral angle restraints derived from TALOS were also implemented (20).

For NMR titration experiments, either ^15^N or ^15^N^13^C-labelled P7 was prepared in the NMR buffer. Unlabelled peptides (β’ NTD or β flap domain tip helix) in the same buffer was introduced at several steps up to a saturating molar excess and 2D ^1^H-^15^N HSQC spectra were recorded at each stage under identical experimental conditions.

For spin labelling the C-terminus of P7 we introduced the spin label S-(2,2,5,5-tetramethyl-2,5-dihydro-1H-pyrrol-3-yl)methyl methanesulfonothioate (MTSL) to the C-terminus of P7 by mutating a V70 to cysteine. The paramagnetic reagent MTSL shortens the transverse relaxation time of nearby nuclei and therefore leads to a loss of peak intensity in the spectrum (21,22).

In the recently described crystal structure of the *Ec* Eσ^70^ [PDB ID 4IGC; (23)] the N-terminal region aa residues from 1 to 8 of the β’ subunit are absent. Furthermore, aa residues 8–11 adopt an extended conformation rather that the helix seen the NMR structure presented. This together with the observation that the fewer aa residues of the β’ N-terminus are modelled in other structures of the *Ec* RNAp suggests some degree of flexibility in this region (24,25). To construct a structural model for the P7-*Ec* Eσ^70^ complex for further analysis, the N-terminus of the β’ subunit was truncated to residue 11 and then extended with our NMR structure of the P7-bound β’ aa residues 1–10. The P7-β’ NTD moiety was rotated as a rigid-body while steric clashes with Eσ^70^ were removed.

### Western blotting

Western blotting experiments were conducted using standard laboratory protocols. *Ec* strain KS1 whole-cell extracts (corresponding to 2.5 × 10^7^ cells) prepared from cells sampled at the end of the β-galactosidase assays were analysed by sodium dodecyl sulphate-polyacrylamide gel electrophoresis gels and proteins were transferred to a Hybond-ECL nitrocellulose membrane using a Trans-Blot Semi-Dry transfer cell (Bio-Rad). Anti-P7 polyclonal antibodies (Eurogentec) and horseradish peroxidase conjugated anti-rabbit IgG (Sigma) in combination with the ECL SuperSignal West Femto Chemiluminescent Substrate kit (Pierce) was used to detect the α-P7 fusion protein. Digital images of the blots were obtained using an LAS-3000 Fuji Imager.

### *In vitro* transcription assays

*In vitro* transcription assays were conducted essentially as previously described (26). Reactions (10 μl) were conducted using final concentrations of 100 nM Eσ^70^, 20 nM *lac*UV5 promoter DNA probe, 0.5 mM dinucleotide primer ApA, 0.05 mM UTP and 3 mCi of [α-^32^P]-UTP. P7 and Eσ^70^ (at a 4:1 molar ratio unless otherwise indicated) were always pre-incubated before the promoter DNA was added to the reaction. The reactions were resolved on a 20% (w/v) urea-denaturing polyacrylamide gel. The gel was visualized and quantified with the use of an FLA-5000 PhosphorImager.

### Native gel mobility assays

All native mobility shift assays were conducted essentially as described previously (26). Binding reactions (10 μl) were set up as described above for the *in vitro* transcription assays. The experiment shown in Supplementary Figure S4B was carried out at ∼4°C essentially as previously described (27).

### Bacterial two-hybrid interaction assays

*LacZ* expression was determined from β-galactosidase assays performed with microtiter plates and a microtiter plate reader as previously described (28). Cultures of *Ec* strain KS1 were grown for 16 h at 30°C in the presence of ampicillin (100 µg/ml), chloramphenicol (30 µg/ml), kanamycin (25 µg/ml) and 0–100 µM Isopropyl β-D-1-thiogalactopyranoside (IPTG). Experiments were conducted at least three times on separate occasions with three biological replicates for each experiment. Similar results were obtained for all experiments. Values shown represent results from a single experiment and the error bars represent standard deviation.

### KMnO_4_ footprinting

Binding reactions were set up as described for the *in vitro* transcription assays in the absence and presence of P7 but using the T5 N25 promoter. The promoter probe for the KMnO_4_ footprinting experiments was formed by mixing equimolar amounts of 100 bp oligonucleotides representing the template and non-template strand sequences of the T5 N25 promoter in a buffer containing 40 mM Tris, pH 7.9, 100 mM NaCl, heating for 2 min at 95°C, and slowly cooling down to 25°C; one of the oligonucleotides was 5′-end-labelled with γ^32^P-ATP to monitor strand-specific oxidation by KMnO_4_. Promoter complexes were treated with 1 mM KMnO_4_ at 37°C for 30 s. Reactions were terminated by the addition of ∼40 μM of 300 mM β-mercaptoethanol followed by ethanol precipitation and 20-min treatment with 10% piperidine at 95°C. Reaction products were next treated with chloroform to remove piperidine, ethanol precipitated, dissolved in 8 μl of urea-formamide loading buffer and resolved in 8% polyacrylamide urea denaturing gel. The A/G ladder was prepared by incubating 40 pmoles of the template DNA probe in 17 μl of ddH_2_O and 50 μl of formic acid for 5 min at room temperature. The DNA was then precipitated by adding 7 μl of 3 M NaOAc and 900 μl of chilled 100% ethanol. The precipitated DNA was dissolved in 90 μl of ddH_2_O, 10 μl of piperidine was added and incubated at 95°C for 10 min. The reaction was chloroform/ethanol precipitated twice; the DNA dissolved in urea-formamide loading buffer and 2–5 μl of this was used for gel loading.

## RESULTS AND DISCUSSION

### The solution structures of P7 and P7-β’ NTD complex

Previous work showed that aa residues 6–9 of the *Xo* β’ subunit are important for the binding of P7 to the *Xo* RNAp (14). To shed light on the mechanism by which P7 inhibits transcription initiation we determined the solution structures of apo P7 and a complex between P7 and *Xo* β’ residues 1–10 (hereafter referred to as the β’ NTD) using multidimensional NMR spectroscopy (Figure 1B-1D, Supplementary Figure S1A, B, Table S3). In the apo P7 structure, the P7 polypeptide folds into a compact globular domain comprising an α-helix packed against a β sheet in a β1 -β2-β3-β4-α1 arrangement (Figure 1B). In the P7-β’ NTD complex β’ NTD folds into a short α-helix between aa residues 4–9 (Figure 1C and D) that positions hydrophobic aa residues L7 and F8 into an exposed hydrophobic cavity between P7 β1 and α1 and in close proximity to P7 residues F50 and V51 (Figure 1C and D). Notably, the carboxyl-terminal ‘tail’ of P7 (residues K65–R73) folds back and L67 also contacts the β’ NTD (Figure 1B–D). Strikingly, in the structure of apo P7 the carboxyl-terminus exhibits multiple conformations within the NMR ensemble that are distinct from the ordered conformation in the P7-β’ NTD complex (Supplementary Figure S1A, B). The rest of the P7 structure is similar in the β’ NTD complex and the free protein. In support of the large-scale conformational changes undergone by P7 on the binding to β’ NTD, we observe dramatic chemical shift perturbations in the 2D ^1^H-^15^N HSQC spectra of P7 saturated with β’ NTD (Supplementary Figure S1C). To provide further evidence for a conformational change in the carboxyl-terminal ‘tail’ of P7 on β’ NTD binding, we introduced the sulfhydryl-specific spin label MTSL to the carboxyl-terminus of P7 by mutating V70 to cysteine. Paramagnetic MTSL shortens the transverse relaxation time of nearby nuclei and leads to a loss of peak intensity in the ^1^H-^15^N HSQC NMR spectrum (21,22). Consistent with the dynamic nature of the carboxyl-terminus of P7, a number of resonances that are widely distributed over the back face of the α1-helix and in the empty hydrophobic binding cavity disappear in MTSL-labelled apo P7 (Supplementary Figure S1D). On the binding to β’ NTD, the reductions in peak intensities are restricted to amides in the immediate vicinity of MTSL labelled position and also include the two labelled leucine amides in β’ NTD (L5 and L7; Supplementary Figure S1D; see ‘Materials and Methods’ section), thus confirming the ordering of the carboxyl-terminus of P7.

To verify the contributions of key aa residues in the P7-β’ NTD interface to the binding of P7 to the β’ subunit of the RNAp, a bacterial-two hybrid (BTH) interaction assay was used. In this assay, a contact between P7 fused to a component of RNAp (here, the α subunit) and β’ NTD fused to a DNA-binding protein (here, the CI protein of bacteriophage λ) activates transcription of a *lacZ* reporter gene under the control of a promoter bearing an upstream λ operator, a recognition site for the CI protein fusion (29). Alanine substitutions at P7 residues F50 and V51 that should interfere with the interaction with β’ NTD based on structural analysis, decreased test promoter activity (Figure 1E). In contrast, the L67A substitution had no effect. Because the intracellular levels of all mutant α-P7 fusion proteins are similar to wild-type α-P7 fusion protein level (Supplementary Figure S1E) the results suggest that aa residues F50 and V51 in P7 are the major determinants for interaction with β’ subunit of the RNAp while the contact with L67 is less important.

### P7 simultaneously interacts with the RNAp β and β’ subunit of the RNAp

**Figure 2.**
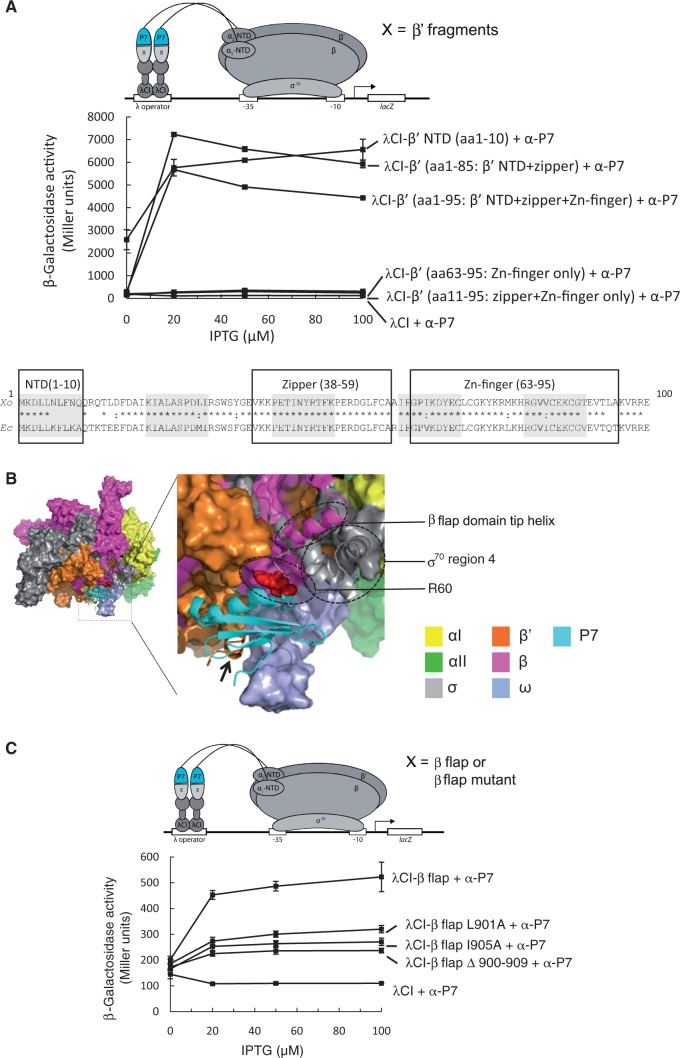

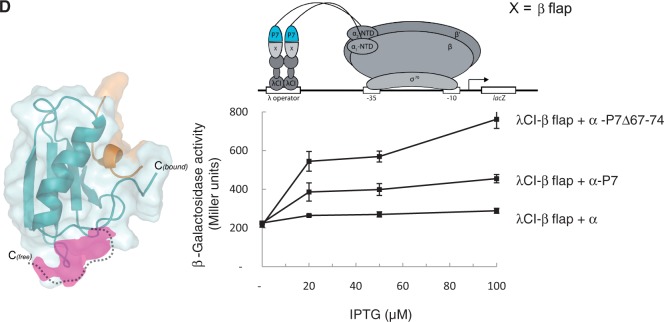
P7 interacts with the β subunit flap domain of the RNAp. (**A**) BTH interaction assay used to detect protein–protein interaction between different fragments of the *Xo* β’ subunit (as indicated and see text for details) and P7. The diagram depicts how the interaction between different fragments of the *Xo* β’ subunit, fused to the bacteriophage λ CI protein (λCI), and P7, fused to the α-NTD (α-P7), activates transcription of the *lacZ* gene. Because the expression of the fusion proteins are under the control of IPTG-inducible promoters, and the cells used for the β-galactosidase assays were grown in the presence of increasing concentrations of IPTG, in the graph the β-galactosidase activity (in Miller units) is expressed as a function of IPTG concentration. The alignment of the aa sequence corresponding to residues 1–100 of the β’ subunit of *Ec* and *Xo* RNAp is shown for comparison with the β’ NTD, zipper and Zn-finger domains boxed. In the alignment, ‘*’ and ‘:’ indicate identical and similar aa residues, respectively. (**B**) Surface representation of the structural model of P7 bound to the *Ec* Eσ^70^. The αI, αII, β, β’, ω and σ^70^ subunits coloured as indicated. The boxed region is enlarged and shows the region around the P7-RNAp interface in more detail. The σ^70^ region 4 and the β flap domain tip helix are shown as cartoon representation and circled, and the black arrow points to the β’ NTD; the side-chain of aa residue R60 of P7 is shown as red surface representation and circled. (**C**) As in (A) but the BTH interaction assay used to detect protein–protein interaction between the *Ec* β flap domain (and mutants thereof, as indicated) and P7. (**D**) Left. Surface representation of free and bound forms of P7 showing the conformational changes in the C-terminus ‘tail’ of P7. The region in P7 unveiled on binding to the β’ NTD is shown in pink. Right. As in (A), but BTH interaction assay is used to detect protein–protein interaction between the *Ec* β flap domain and P7 and a truncated P7 mutant lacking the C-terminal ‘tail’ (P7Δ67–74).

As two functionally important structural elements of the bacterial RNAp, the Zn-finger (*Xo* β’ aa 63–95) and the β’ zipper (*Xo* β’ aa 38–59) are adjacent to the β’ NTD (30,31), we used the BTH interaction assay to assess whether these additional regions of *Xo* β’ contribute to P7 binding. Accordingly, we measured if P7 can interact with different fragments of *Xo* β’ comprising aa residues 1–85 (contains the β’ NTD and the β’ zipper), 1–95 (β’ NTD, the β’ zipper domain, and the Zn-finger), 11–95 (contains only the β’ zipper domain and the Zn-finger element) and 63–95 (contains only the Zn-finger). The BTH construct containing the β’ NTD served as a positive control. The results show that the β’ NTD is sufficient for the binding of P7 to the RNAp (Figure 2A). Because the interaction between P7 and the β’ NTD alone is stronger than the interaction between P7 and the β’ fragment containing the β’ zipper and Zn-finger domains (fragment comprising aa residues 1–95), we suggest that these domains do not contribute significantly to the binding of P7 to the RNAp. Consistent with this view, no interaction between fragments comprising aa residues 63–95 (Zn-finger only) and 11–95 (zipper and Zn-finger only) is detected. We then derived a structural model for the P7-Eσ^70^ complex by extending the β’ subunit of the recently described crystal structure of the *Ec* Eσ^70^ (23) with our P7-β’ NTD solution structure (see ‘Materials and Methods’ section). In the model, P7 is adjacent to the β flap domain and region 4 of σ^70^ (Figure 2B), suggesting that P7 could interact with the β flap domain and/or region 4 of σ^70^. We reasoned that this interaction, if present, should occur with the *Ec* RNAp, as a hybrid *Ec*-based RNAp (containing the β’ NTD, i.e. aa residues 1–10 of the *Ec* β’ subunit substituted with corresponding aa residues of the *Xo* β’ subunit; hereafter referred to as ^P7S^Eσ^70^) binds P7 and is readily inhibited by P7 in the same way as *Xo* Eσ^70^ (14). We therefore used the BTH interaction assay to determine if P7 interacts with the β flap and/or σ^70^ region 4 domains using the *Ec* β flap domain aa residues 831–1057 and *Ec* σ^70^ domain 4 residues 528–613 fused to bacteriophage λ CI protein. The results show that P7 indeed interacts with *Ec* β flap domain (Figure 2C), while there is little or no interaction of α-P7 with λCI fused to *Ec* σ^70^ region 4 (Supplementary Figure S2A). The specificity of the interaction between P7 and the β flap domain is demonstrated by its abrogation when aa residues corresponding to the β flap domain tip helix (aa residues 900–909) are deleted or alanine substitutions in the β flap domain tip helix are introduced (L901A and I905A) (Figure 2C). Because the removal of carboxyl-terminus aa residues 67–74 of P7 markedly increases the ability of P7 to interact with the β flap domain in the context of the BTH assay (Figure 2D) [even though the relative protein levels of wild-type P7 and P7Δ67–74 are comparable under conditions of the BTH assay (Supplementary Figure S2B)], the folding back of the carboxyl-terminal ‘tail’ of P7 on binding to the β’ NTD could unveil a β flap interaction surface, which comprises aa residues V11, L34 and R60 (Figure 2D and see later). In summary, the results to a large extent confirm the structural model of the *Ec* RNAp-P7 complex and suggest that the binding of P7 to the β’ NTD is necessary before the interaction between P7 and the β flap domain tip helix of the RNAp can take place.

### P7-β flap interaction is important for transcription inhibition

**Figure 3.**
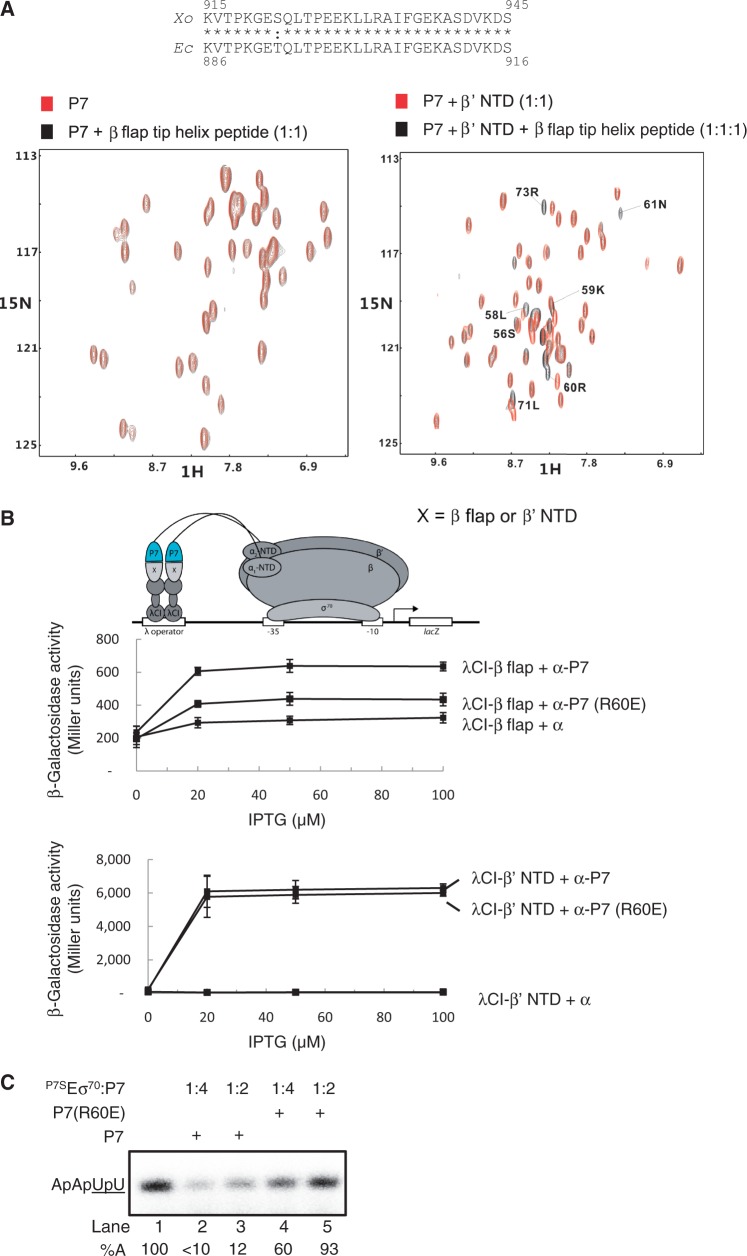
P7-β flap interaction is important for transcription inhibition. (**A**) ^1^H-^15^N-HSQC NMR spectra of either ^15^N,^13^C-labelled P7 (left) or the p7- β’ NTD complex (right) in the presence and absence of the β flap domain tip helix peptide based on *Xo* β flap domain aa sequence. The aa residues significantly affected in P7 are labelled. The alignment of the aa sequence corresponding to the β flap domain tip helix from the *Ec* and *Xo* β subunit is shown for comparison. In the alignment, ‘asterisk’ and ‘colon’ indicate identical and similar aa residues, respectively. (**B**) BTH interaction assay used to detect protein–protein interaction between the β’ NTD or *Ec* β flap domain and P7 and P7 (R60E) mutant. The diagram depicts how the interaction between the β’ NTD or *Ec* β flap domain, fused to the bacteriophage λ CI protein (λCI), and P7 and P7 (R60E), fused to the α-NTD (α-P7), activates transcription of the *lacZ* gene. Because the expression of the fusion proteins (λCI-β’ NTD and flap λCI-β flap and α-P7) are under the control of IPTG-inducible promoters, and the cells used for the β-galactosidase assays were grown in the presence of increasing concentrations of IPTG, in the graphs the β-galactosidase activities (in Miller units) are expressed as a function of IPTG concentration. (**C**) Autoradiograph of 20% (w/v) denaturing urea gel showing the synthesis of ApApUpU transcript (underlined nucleotides are α^32^P labeled) from the *lac*UV5 promoter by ^P7S^Eσ^70^ in the absence (lane 1) and presence of P7 (lanes 2 and 3) and P7 (R60E) (lanes 4 and 5). The percentage of ApApUpU transcript synthesized (%A) in the reactions with P7 with respect to reactions without P7 is normalized to unincorporated [α-^32^P]-UTP (not shown) and given at the bottom of the gel for each reaction. All values for %A obtained from at least three independent experiments fell within 5% of the value shown.

To analyse the β flap-P7 interaction in more detail we performed NMR chemical shift mapping with a synthetic peptide spanning the β flap tip helix of the *Xo* β flap domain (*Xo* aa residues (915–945; Figure 3A). While an interaction between apo P7 and the *Xo* β flap tip peptide could not be observed (Supplementary Figure S3A), the *Xo* β flap tip peptide-dependent backbone amide chemical shift changes in P7 are detected when fully saturated with the β’ NTD (Figure 3A). This observation is consistent with the view that the binding of P7 to the β’ NTD facilitates the interaction between P7 and the β flap domain tip helix of the RNAp (see above). Several of the perturbed residues (S56, L58, K59, R60, N61, L71 and R73) map to the surface on P7 that is unveiled on the binding to the β’ NTD (Figure 3A) and four of these residues (L58, K59, R60 and N61) are proximal to the β flap domain tip helix in our structural model (Figure 2B). Consistent with this observation, mutant P7 harbouring a charge reversal substitution at aa residue R60 (R60E) reduces the ability of P7 to interact with the β flap domain, but not with the β’ NTD in the BTH interaction assay (Figure 3B). The interaction with the β flap domain is important for the ability of P7 to function as a transcription inhibitor because mutant P7 harbouring the R60E substitution displays a reduced ability to inhibit the transcription initiation by ^P7S^Eσ^70^ from the *lac*UV5 promoter, a well-characterized −35/−10 class of bacterial promoter (Figure 3C).

### P7 displaces σ^70^ from the RNAp

**Figure 4.**
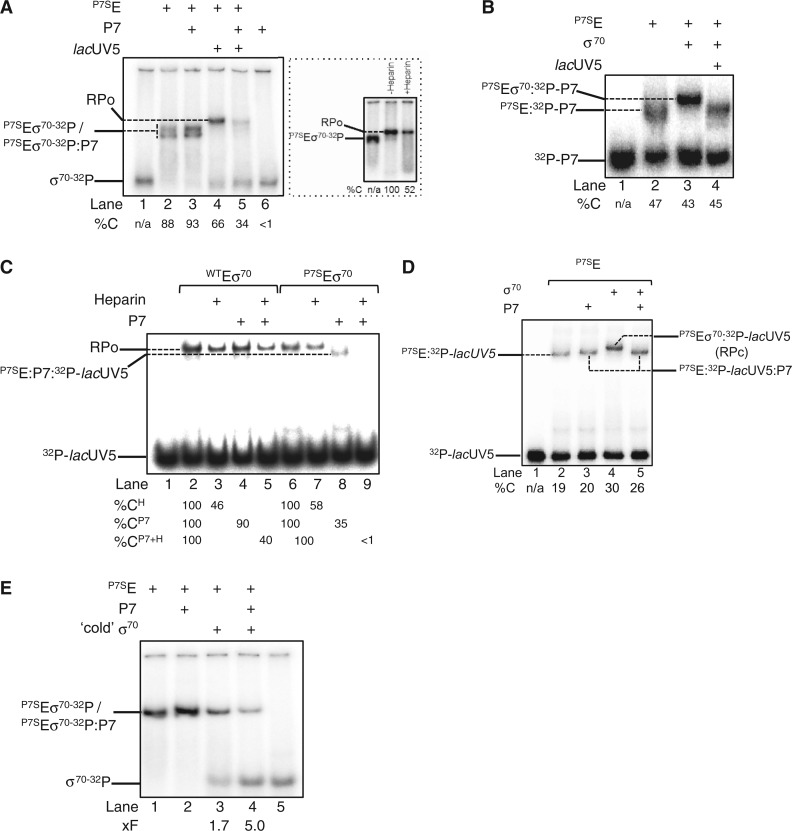
P7 displaces σ^70^ from the RNAp. (**A**) Autoradiograph of a 4.5% (w/v) native polyacrylamide gel showing results from experiments conducted with σ^70-32^P to determine if P7 causes the displacement of σ^70^ from the RNAp. The %C indicates the radioactivity in the different complexes as a percentage of free σ^70-32^P in lane 1 (please note that the sum of total radioactivity in all lanes except lane 1 is equal because the Eσ^70-32^P poorly enters the gel matrix under our experimental conditions). The inset shows an autoradiograph of a 4.5% (w/v) native polyacrylamide gel demonstrating that the slower migrating complex that forms when promoter DNA is added to the ^P7S^Eσ^70-32^P is resistant to heparin and thus represents the RPo. The percentage ^P7S^Eσ^70-32^P-*lac*UV5 complexes (i.e. RPo) remaining following challenge with heparin compared with reactions with no heparin added is given at the bottom of the gel. (**B**) As in (A), but experiments were conducted with ^32^P-P7. The %C indicates the radioactivity in the different complexes as a percentage of free ^32^P-P7 in lane 1. (**C**) As in (A), but experiments were conducted with ^32^P-*lac*UV5 promoter DNA and wild-type RNAp was used as a negative control in lanes 2–5. The %C^H^ indicates the relative percentages of ^WT^Eσ^70^:^32^P-*lac*UV5 and ^P7S^Eσ^70^:^32^P-*lac*UV5 complexes (i.e. RPo) remaining following challenge with heparin compared with reactions with no heparin added (lanes 1 and 6, respectively). The %C^P7^ indicates the relative percentages of promoter complexes formed in the presence of P7 compared with reactions with no P7 added (lanes 1 and 6, respectively). The %C^P7+H^ indicates the relative percentages of heparin-resistant promoter complexes formed in the presence of P7 compared with reactions with no P7 or heparin added (lanes 1 and 6, respectively). (**D**) As in (C), but experiments were conducted at 4°C and only with the P7-sensitive RNAp (see text for details). The %C indicates the radioactivity in the different complexes as a percentage of free ^32^P-*lac*UV5 in lane 1. (**E**) As in (A), but ^P7S^Eσ^70-32^P and ^P7S^Eσ^70-32^P:P7 complexes were challenged with x5-fold molar excess of non-radioactive ‘cold’ σ^70^ in lanes 3 and 4. The fold decrease (%F) in the intensity of the radioactive signal originating from σ^70-32^P in ^P7S^Eσ^70-32^P and ^P7S^Eσ^70-32^P:P7 complexes in the presence of ‘cold’ σ^70^ relative to complexes formed in its absence (lanes 1 and 2) are given at the bottom of the gel. In (A–E) the components present in each lane are indicated on the top of each gel and the identity of the different protein–protein and protein–DNA complexes are indicated. In (A–E) representative results from at least three independent experiments are shown and the percentages/fold differences calculated represent averages obtained from three independent experiments and fell within 3–5% of the percentage shown.

The results so far establish that P7 can simultaneously interact with the β’ NTD and the β flap domain tip helix of the RNAp. Based on an observation that RNAp purified from *Xo* cells infected by Xp10 phage is largely devoid of the σ^70^ (compared with RNAp isolated from uninfected cells) (32) and the functionally obligatory role the β flap domain plays during Eσ^70^ formation (5), we hypothesized that P7 could inhibit transcription by destabilizing the interaction between σ^70^ and RNAp. Initially, we probed directly whether P7 causes σ^70^ displacement using an electrophoretic mobility shift assay (EMSA) with σ^70-32^P. As shown in Figure 4A, the addition of P7-sensitive core RNAp (^P7S^E) to σ^70-32^P results in a slower migrating radioactive complex that represents the RNAp holoenzyme (^P7S^Eσ^70-32^P) (compare lanes 1 and 2). The addition of P7 to ^P7S^Eσ^70-32^P does not result in any detectable changes in the mobility of the ^P7S^Eσ^70-32^P or any obvious decrease in the intensity of the radioactive signal originating from the ^P7S^Eσ^70-32^P (in fact an intensification of the radioactive signal in the ^P7S^Eσ^70-32^P complex was observed) and both the ^P7S^Eσ^70-32^P and ^P7S^Eσ^70-32^P:P7 complexes run as a doublet on the native gel. Thus, the results suggest that P7 does not displace the σ^70^ from the RNAp (Figure 4A, lane 2 and 3). The addition of *lac*UV5 promoter probe to ^P7S^Eσ^70-32^P results in a new complex that migrates slower than the ^P7S^Eσ^70-32^P complexes (Figure 4A, lane 4). The new complex represents the RPo because it is resistant to the polyanion heparin [heparin-resistance is a hallmark feature of the RPo formed on the *lac*UV5 promoter (Figure 4A, inset)]. However, when the *lac*UV5 promoter probe is added to the ^P7S^Eσ^70-32^P:P7 complex, the radioactivity originating from the band corresponding to the RPo decreases by ∼50%, thus indicating the displacement of σ^70-32^P from the RNAp in the presence of P7 (Figure 4A, compare lane 4 and 5). Additional experiments with ^32^P-P7 confirm that P7 displaces σ^70^ from ^P7S^Eσ^70^:P7 complex on interaction with the *lac*UV5 promoter probe: As expected, ^32^P-P7 and σ^70^ can bind to the RNAp at the same time (Figure 4B, lanes 1–3), but the addition of the *lac*UV5 promoter probe to the ^P7S^Eσ^70^:^32^P-P7 results in a complex that migrates at the identical position on the native gel as the ^P7S^E:^32^P-P7 complex (Figure 4B, compare lanes 2–4). Further experiments with ^32^P-*lac*UV5 promoter probe corroborate the view that P7 causes the displacement of σ^70^ from the RNAp on interaction with DNA: Results shown in Figure 4C reveal that the presence of P7 results in a faster-migrating (relative to the RPo) and heparin-sensitive complex that is likely composed of P7, core RNAp subunits and *lac*UV5 (Figure 4C, lanes 6–9; Figure 4B, lane 4; Figure 4A, lane 5). To independently verify that P7 displaces σ^70^ from the RNAp, which indeed results in the formation of a non-specific and thus transcriptionally inactive complex between RNAp core and DNA, we analysed promoter complexes formed on the σ^70^-dependent T5 N25 promoter by potassium permanganate (KMnO_4_) probing. KMnO_4_ is a single-stranded thymine-reactive DNA oxidizing agent that is widely used to detect σ^70^-dependent local DNA melting around and downstream of the consensus −10 element on promoters by the RNAp that is indicative of RPo formation. Whereas KMnO_4_-mediated DNA cleavage is seen on the unpaired thymine at positions −12 to −3 on the non-template and −4 on the template strands by ^P7S^Eσ^70^, as expected, no DNA cleavage at these positions on either the non-template on template strands of the T5 N25 promoter is detected in reactions containing P7, which is indicative of a non-specific and thus transcriptionally inactive complex between the promoter and core RNAp (Supplementary Figure S4).

Next, we repeated the EMSA experiments with ^32^P-*lac*UV5 at ∼4°C to determine if P7 causes σ^70^ dissociation on initial engagement of RNAp with the promoter (i.e. during RPc formation) or during the RPo formation. Recall that the majority of promoter complexes formed on the *lac*UV5 promoter at ∼4°C correspond to RPc (33). As shown in Figure 4D, the complex formed in the presence of P7 and *lac*UV5 promoter (lane 5) has an identical mobility as the ^P7S^E:^32^P-*lac*UV5:P7 complex (lane 3) and migrates faster than the ^P7S^Eσ^70^:^32^P-*lac*UV5:P7 (RPc) complex (lane 4). This result suggests that P7-induced displacement of σ^70^ from the RNAp occurs at the early stages of engagement of promoter DNA by the RNAp holoenzyme.

Collectively, the results indicate that P7 simultaneously interacts with the β’ and β subunits of the bacterial RNAp and inhibits transcription by σ^70^ displacement when RNAp attempts to interact with the promoter. P7 first docks onto the accessible β’ NTD and positions itself proximal to the β flap domain. A new interaction surface is unveiled on P7 that interfaces with the β flap domain tip helix, thereby (i) interfering with σ^70^ region 4/β flap interaction and (ii) weakening the interaction between σ^70^ and core RNAp. The latter effect apparently results in the dissociation of σ^70^ when RNAp undergoes additional conformational changes while engaging promoter DNA (see below). Results from the following observations support this scenario: Under identical experimental parameters of the BTH assay, the binding of σ^70^ region 4 to the β flap domain is ∼4 - to 5-fold weaker than the interaction between P7 and the β flap domain (5), and the interaction between P7 and β flap is important for the mode of transcription inhibition by P7 because mutant P7 harbouring the R60E mutation displays significantly compromised (by ∼50%) ability to interact with the β flap and inhibit transcription initiation by ^P7S^Eσ^70^ (Figure 3B and C). However, the interface between σ^70^ and RNAp is extensive in which the interaction between σ^70^ region 2 and the β’ coiled-coiled motif represents the major interaction interface (34), whereas the interface between σ^70^ region 4 and the β flap is relatively weak. Therefore, it is a formal possibility that P7 binding to the RNAp adversely affects the affinity of σ^70^ to the RNAp. In support of this view, σ^70-32^P dissociates much more readily from the RNAp on challenging the Eσ^70-32^P with ‘cold’ unlabeled σ^70^ in the presence of P7 than in its absence (Figure 4E), thus indicating that P7 contributes to weaken the interaction between σ^70^ and core RNAp, which is evidently amplified in the presence of promoter DNA. In summary, our results provide a detailed molecular mechanism that explains the previous observation that RNAp isolated from Xp10 infected cells is largely devoid of σ^70^ compared to RNAp isolated from uninfected cells (32).

## CONCLUSION

Many bacteria and phages encode a specialized class of proteins, called anti-σ factors, to modulate transcription by controlling the availability and activity of σ-factors (35). Anti-σ factors interact with their cognate σ-factors to either inhibit their activity or, as in the case of the T4 phage encoded protein AsiA, alter the promoter-specificity of the bacterial RNAp (36). Unlike other phage-encoded bacterial transcription initiation inhibitors described to date, which function through direct interactions with σ^70^ (26), our results provide no evidence for an interaction between P7 and σ^70^. However, it seems that P7 functions as a type of anti-σ factor by inhibiting transcription initiation by σ^70^ displacement on engagement of the RNAp with the promoter DNA.

Because many phage-encoded antiterminators, such as λ phage proteins N and Q (37,38) and *Thermus thermophilus* phage P23-45 Gp39 protein (39), interact with the β flap domain, it is conceivable that the antitermination function of P7, like its transcription initiation inhibition function, also requires interactions with the β flap domain. In conclusion, this study not only underscores the β flap as a nexus for regulation of the bacterial RNAp by bacterial and phage transcription regulators, but also highlights the remarkable variation and efficiency in the design of phage-encoded bacterial RNAp inhibitors and thus serves to inspire novel ways for developing antibacterial drugs targeting the bacterial RNAp based on strategies used by bacteriophages.

## SUPPLEMENTARY DATA

Supplementary data are available at NAR Online.

## FUNDING

Biotechnological and Biological Research Council (to S.W. and S.M.) and Wellcome Trust Investigator awards (to S.W. and S.M.). Funding for open access charge: Imperial College (through Wellcome Trust and BBSRC open access policy).

*Conflict of interest statement*. None declared.

## Supplementary Material

Supplementary Data
