# Supplementary material for: A bacteriophage transcription regulator inhibits bacterial transcription initiation by σ-factor displacement
Source: Nucleic Acids Res. 2014 Jan 28;42(7):4294–305. doi: 10.1093/nar/gku080 (PMC3985653; doi:10.1093/nar/gku080)
Supplement: Supplementary Data [file supp_42_7_4294__index.html]

A bacteriophage transcription regulator inhibits bacterial transcription initiation by σ-factor displacement — A bacteriophage transcription regulator inhibits bacterial transcription initiation by σ-factor displacement — Supplementary Data 

# A bacteriophage transcription regulator inhibits bacterial transcription initiation by σ-factor displacement

## Supplementary Data

files

**Files in this Data Supplement:**

- Supplementary Data - pdf file
