# Supplementary material for: A bacteriophage transcription regulator inhibits bacterial transcription initiation by σ-factor displacement
Source: Nucleic Acids Res. 2014 Jan 28;42(7):4294–305. doi: 10.1093/nar/gku080 (PMC3985653; doi:10.1093/nar/gku080)
Supplement: Supplementary Data [file supp_gku080_nar-03303-m-2013-File006.pdf]

Supplemental Information

A bacteriophage transcription regulator inhibits bacterial transcription initiation by σ-factor displacement

Bing Liu[1,3], Andrey Shadrin[1,3], Carol Sheppard[1], Vladimir Mekler[2], Yingqi Xu[1], Konstantin Severinov[2], Steve Matthews[1,*] and Sivaramesh Wigneshweraraj[1,*]

[1]MRC Centre for Molecular Microbiology and Infection, Imperial College London, SW7 2AZ, UK and [2]Waksman Institute for Microbiology and Department of Molecular Biology and Biochemistry; Rutgers, The State University of New Jersey; Piscataway, NJ USA.

[3]The authors wish it to be known that, in their opinion, the first two authors should be regarded as joint First Authors

* To whom correspondence should be addressed. Tel: +44 (0) 207 594 1867; Fax: +44 (0) 207 594 3055; E-mail: s.r.wig@imperial.ac.uk. Correspondence may also be addressed to s.j.matthews@imperial.ac.uk.

Figure S1

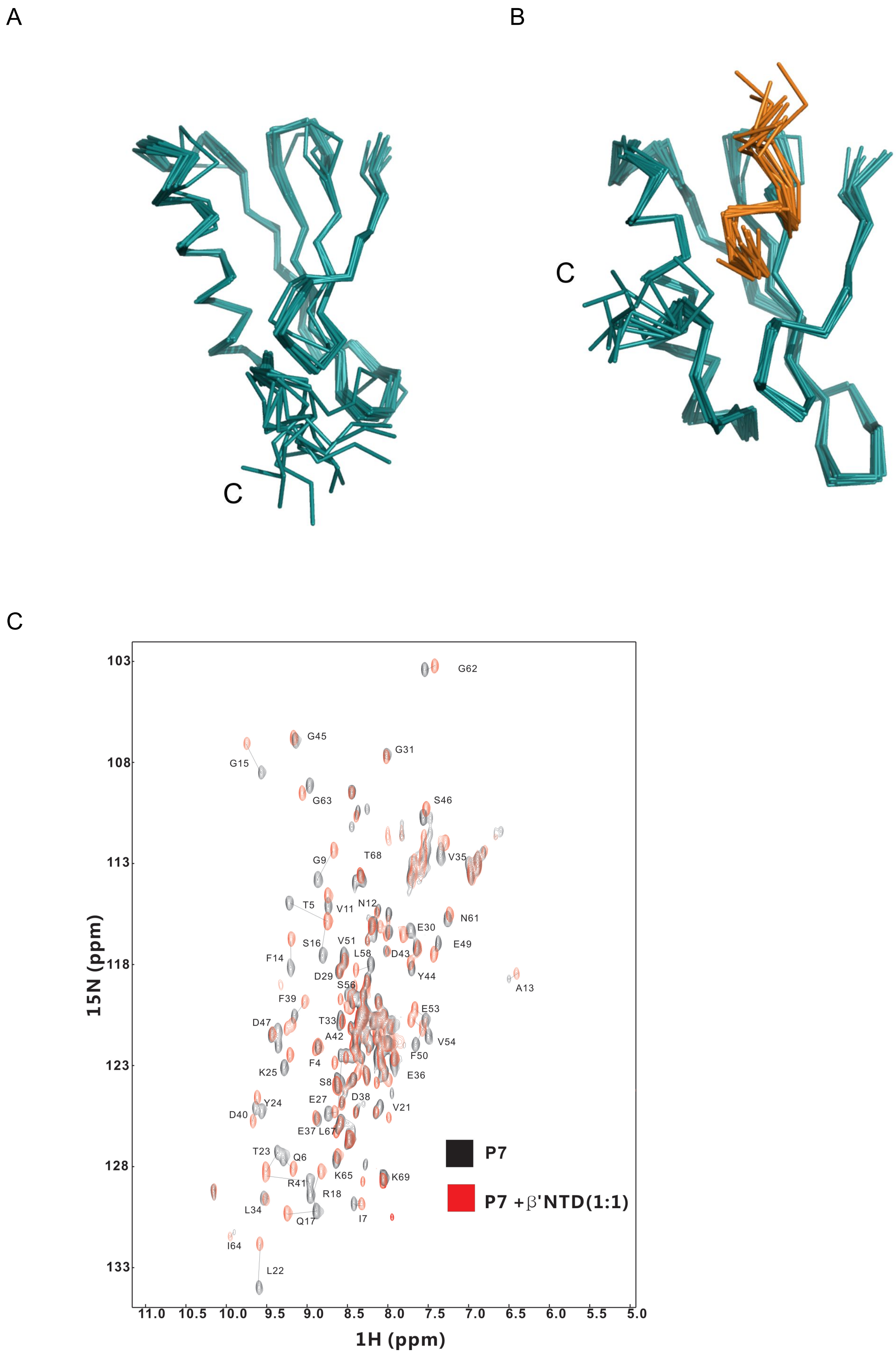
A
B
C
C
C
103
108
113
118
123
128
133
15N (ppm)
11.0 10.5 10.0 9.5 9.0 8.5 8.0 7.5 7.0 6.5 6.0 5.5 5.0
1H (ppm)
G62
G45
G31
G15
G63
S46
G9
T68
V35
T5
V11
N12
E30
N61
S16
V51
L58
D43
E49
F14
D29
Y44
A13
S56
F39
E53
D47
T33
A42
V54
F50
F4
K25
E36
S8
E27
D38
V21
Y24
D40
E37
L67
T23
Q6
K65
K69
R41
R18
L34
Q17
I7
I64
L22
P7
P7 +β'NTD(1:1)

Figure S1 (contd.)

D

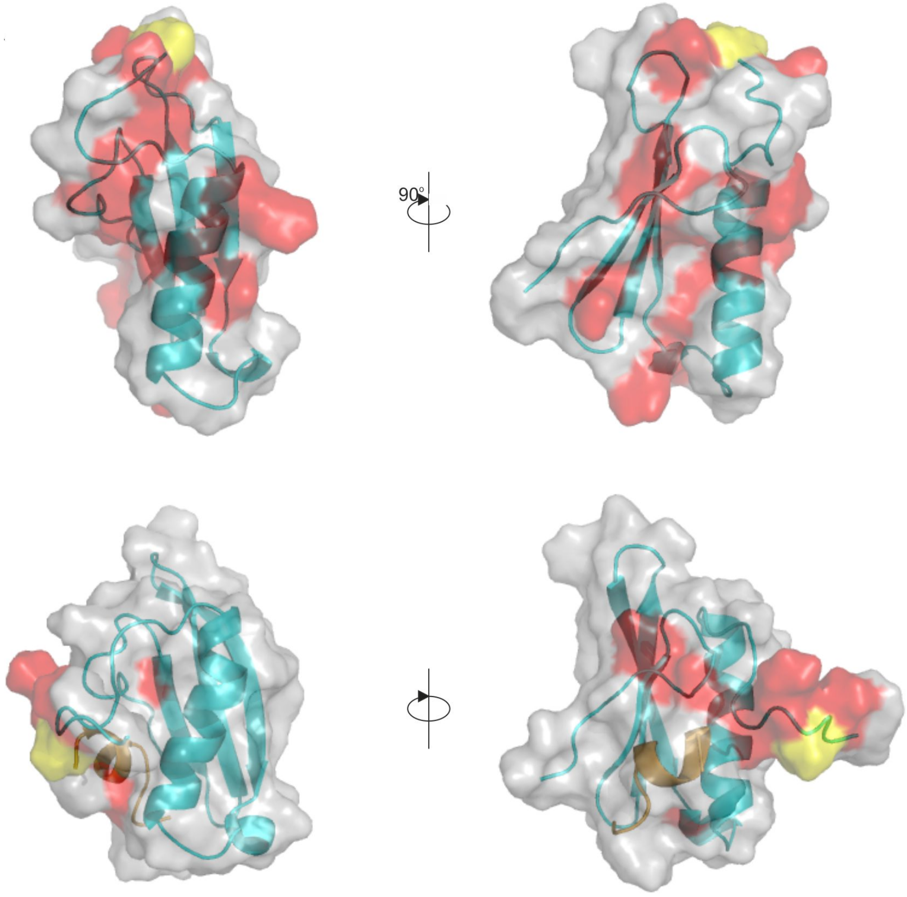

E

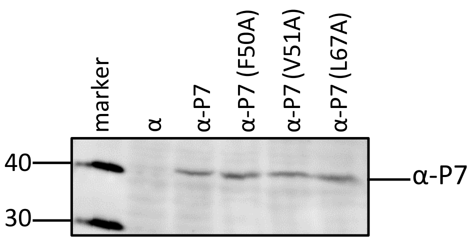

Fig. S1. The solution structures of P7 and P7- β' NTD complex. (A) The final family of NMR structures from the structure calculation of the solution structure of P7. (B) The final family of NMR structures from the structure calculation of the P7-β' NTD complex. In (A) and (B) the C-terminus of P7 is indicated. (C) Overlay of 2D ^{1}H-^{15}N HSQC spectra of ^{15}N-^{13}C-P7 in the presence (red) and absence (black) of β' NTD recorded at pH 8.7, 283 K. Selected resonance assignment are indicated. (D) Molecular surface of P7 color-coded according to paramagnetic relaxation enhancement (PRE). Front and back view of free P7 (top) and P7-β' NTD complex (bottom). Amide resonances for residues broadened beyond detection are shown in red, which includes both L5 and L7 of the β' NTD peptide, and the spin labelled C-terminal residue (V70) in shown in yellow. (E) Western blot analysis to assess intracellular levels of the α-P7 fusion proteins probed using polyclonal anti-P7 antibodies. The results rule out the possibility that the failure of the mutant α-P7 fusion protein to interact with β' NTD is attributable to protein instability.

Figure S2

A

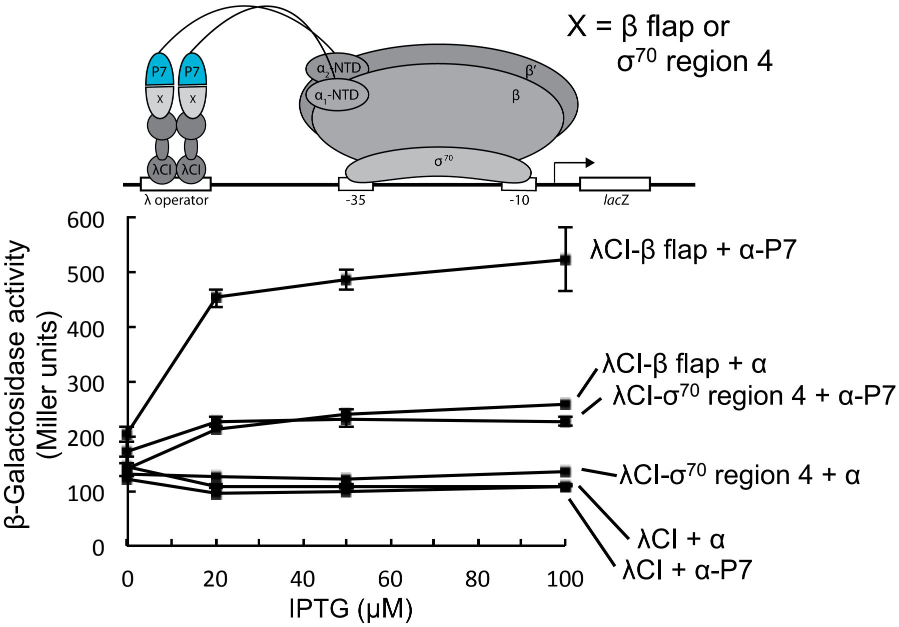

B

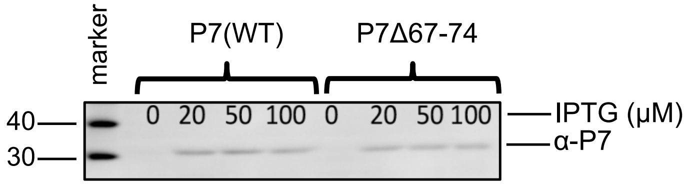

Fig. S2. P7 interacts with the β subunit flap domain of the RNAp. (A) BTH interaction assay used to detect protein-protein interaction between the *Ec* σ^{70} region 2 or 4 and P7. The diagram depicts how the interaction between *Ec* σ^{70} region 4, fused to the bacteriophage λ CI protein (λCI), and P7, fused to the α-NTD (α-P7), activates transcription of the *lacZ* gene. Since the expression of the fusion proteins (λCI-σ^{70} region 4 and α-P7) is under the control of IPTG-inducible promoters, the cells used for the β-galactosidase assays were grown in the presence of increasing concentrations of IPTG and in the graph the β-galactosidase activity (in Miller units) is expressed as a function of IPTG concentration. The activity curves for the relevant control

combination of plasmids are also shown. (B) Western blot analysis to assess intracellular levels of the α-P7 fusion proteins probed using polyclonal anti-P7 antibodies. The results rule out the possibility that the increased activity of P7Δ67-74 to interact with *Ec* β flap domain is attributable to increased level of mutant protein expression.

Figure S3

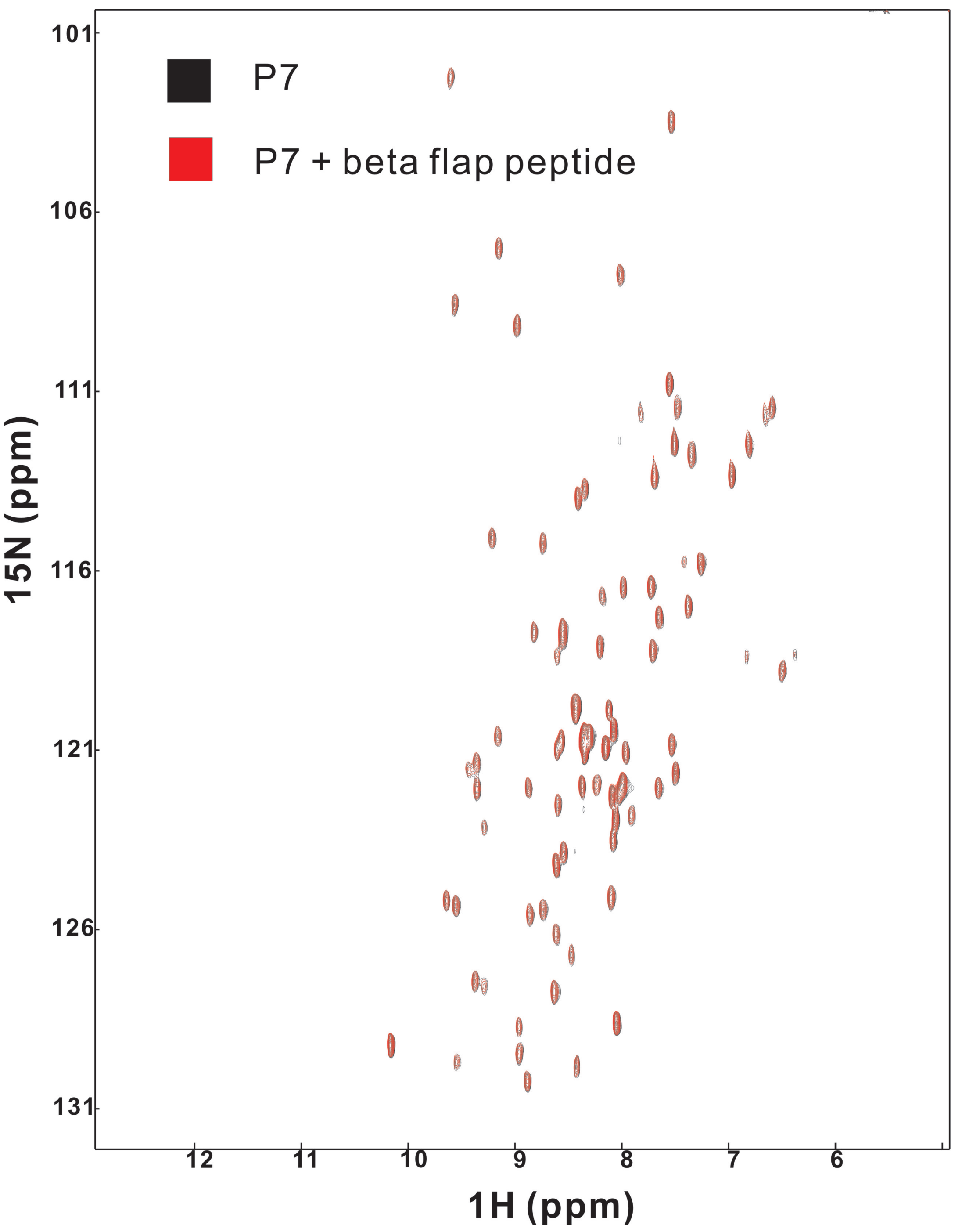

Fig. S3. P7-β flap interaction is important for transcription inhibition. Overlay of 2D ^{1}H-^{15}N HSQC spectra of ^{15}N-^{13}C-labelled P7 in the presence (red) and absence (black) of β flap domain tip helix peptide. The NMR spectra display no chemical shift perturbations.

Figure S4

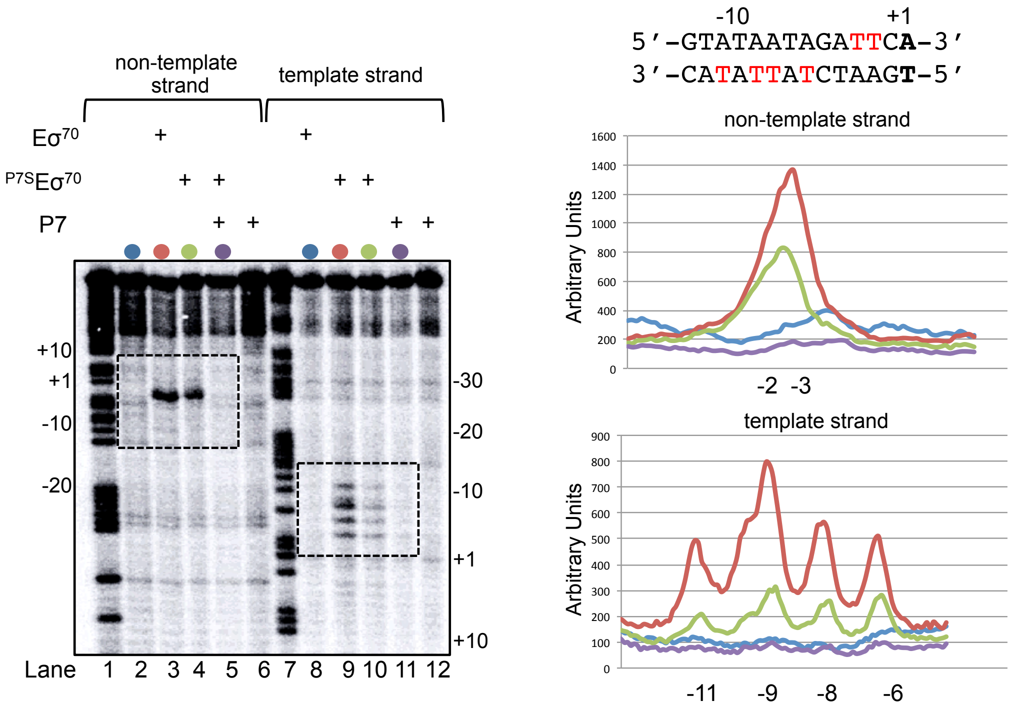

Fig. S4. P7 displaces σ[70] from the RNAp. An Autoradiograph of an 8% (w/v) urea-denaturing polyacrylamide gel showing DNA melting in the RPo and absence of DNA melting in promoter complexes formed in the presence of P7. Lanes 1 and 2 contain the GA marker ladder, which was generated using T5 N25 promoter probe in which the 5' ends of the non-template and template DNA strands, respectively, are ^{32}P-end-labelled. The reaction components in the other lanes are indicated at the top of the gel. To the right of the gel, lane profile traces of the boxed region from lanes 2-5 and lanes 8-11) are shown, and the positions of the $KMnO_4$-reactive thymines are indicated and reactions colour coded as shown. The promoter sequence of the T5 N25 promoter from positions −13 to +1 is shown at the top of the graphs: $KMnO_4$-reactive thymines are shown in red for each DNA strand and the transcription start site (at +1) is shown in bold typeface. The result shown is a representative image from two independent experiments.

Table S1. DNA oligonucleotides used in this work

#	Sequence, 5' --> 3'	Application
1	ggaattc*catATG*aacgaattcacccagat	Cloning of P7 gene into pET33b(+), contains NdeI site on 5' end
2	cgc*ggatcc*TCAtcgggtaagcaccttggt	Cloning of P7 gene into pET33b(+) and pBRα, contains BamHI site on 5' end
3	ttt*gcggccgc*aATGaacgaattcacccagat	Cloning of P7 gene into pBRα, contains NotI site on 5' end
4	ttt*ggatcc*TTATCAtcgggtaagcaccttggt	Cloning of P7 gene into pBRα, contains BamHI site on 5' end
5	ttt*ggatcc*TTAgtccttgatgccgccgtt	Cloning of truncated P7Δ67-74 (1-66aa) gene into pBRα, contains BamHI site on 5' end
6	*ggccgc*aATGaaagacctgctcaacctcttcaatcagTGATAA*g*	Cloning of *Xo* RNAp β'NTD(aa1-10) fragment between NotI and BamHI restriction sites of pACλCI
7	*gatcc*TTATCActgattgaagaggttgagcaggtctttCATt*gc*	Cloning of *Xo* RNAp β'NTD(aa1-10) fragment between NotI and BamHI restriction sites of pACλCI
8	tatat*gcggccgc*aATGaaagacctgctcaacctcttc	Cloning of *Xo* RNAp β' subunit fragments beginning at 1aa into pACλCI, contains NotI site on 5' end
9	tatat*gcggccgc*aCAGcgccagacgctg	Cloning of *Xo* RNAp β' subunit fragments beginning at 11aa into pACλCI, contains NotI site on 5' end
10	tatat*gcggccgc*aGGAccgatcaaggactacgaatg	Cloning of *Xo* RNAp β' subunit fragments beginning at 63aa into pACλCI, contains NotI site on 5' end
11	tatat*ggatcc*TTATCAggccagggtgacttcg	Cloning of *Xo* RNAp β' subunit fragments ending at 95aa into pACλCI, contain BamHI site and stop codons on 5' end
12	gaag*ggatcc*TTAgcagaccacgccacggtgc	Cloning of *Xo* RNAp β' subunit fragments ending at 85aa into pACλCI, contains BamHI site and stop codon on 5' end
13	*gacgacgacaag*ATGaacgaattcacccag	Cloning of P7 gene into pET46, contains specific sequence for ligase-independent cloning on 5' end
14	*gaggagaagcccggTTA*TCAtcgggtaagcaccttgg	Cloning P7 gene into pET46, contains specific sequence for ligase-independent cloning and stop codons on 5' end
15	cggcggcatcaaggacctgaccaag**TGC**cttacccgatgaccgggc	SDM to incorporate V70C amino acid substitution in P7 gene in pET46:P7 (GAG->TGC)
16	gcccggtcatcgggtaag**GCA**cttggtcaggtccttgatgccgccg	SDM to incorporate V70C amino acid substitution in P7 gene in pET46:P7 (GAG->TGC)
17	gcagcgatcccgaa**GC**Cgtggcagaggtc	SDM to incorporate F50A amino acid substitution in P7 gene (AAC->GCC)
18	gacctctgccacg**GC**Ttcgggatcgctgc	SDM to incorporate F50A amino acid substitution in P7 gene (AAC->GCC)
19	gcgatcccgaattcG**C**Ggcagaggtcagctc	SDM to incorporate V51A amino acid substitution in P7 gene (GTG->GCG)
20	gagctgacctctgcC**G**Cgaattcgggatcgc	SDM to incorporate V51A amino acid substitution in P7 gene (GTG->GCG)
21	gtcagctcgtatctcaag**GA**Gaacggcggcatcaagg	SDM to incorporate R60E amino acid substitution in P7 gene (AGG>GAG)
22	ccttgatgccgccgttC**TC**cttgagatacgagctgac	SDM to incorporate R60E amino acid substitution in P7 gene (AGG>GAG)
23	gcggcatcaaggac**GC**Gaccaaggtgcttac	SDM to incorporate R67A amino acid substitution in P7 gene (CTG>GCG)
24	gtaagcaccttggtC**GC**gtccttgatgccgc	SDM to incorporate R67A amino acid substitution in P7 gene (CTG>GCG)
25	gttagctcactcattaggcaccccaggc**TTTACA**ctttatgcttccggctcg**TATAAT**gtgtgg **A**attgtgagcggataacaatttcacacaggaaacag	Used for reconstitution of lacUV5 promoter [-64+36], non-template strand
26	ctgtttcctgtgtgaaattgttatccgctcacaattccacacattatacgagccggaagcataaagt gtaaagcctggggtgcctaatgagtgagctaac	Used for reconstitution of lacUV5 promoter [-64+36], template strand
27	tctttgctcaaagaatcataaaaaatttat**TTGCTT**tcaggaaaatttttctg**TATAAT**agatt c**A**taaatttgagagaggagtttaaatatggctggttc	Used for reconstitution of T5N25 promoter [-65+35], non-template strand
28	gaaccagccatatttaaactcctctctcaaatttatgaatctattatacagaaaaattttcctgaaa gcaaataaatttttttatgattctttgagcaaaga	Used for reconstitution of T5N25 promoter [-65+35], template strand

Sequences corresponding to restriction endonucleases sites are italicised and underlined. Codons encoding to first amino acids of target proteins as well as stop-codons indicated in uppercase. In oligonucleotides used for site directed mutagenesis (SDM) the mutated codon is indicated in uppercase and nucleotides changed highlighted in bold. In oligonucleotides used for promoter DNA reconstitution, the -35 and -10 promoter elements as well as transcription start shown in bold uppercase; parts corresponding to mRNA sequence are underlined.

Table S2. Plasmids used in this work.

Name	Resistance	Relevant Characteristics	Reference
pBRα	Amp	Encodes α-NTD (residues 1-248) of the α-subunit of *Ec* RNAp under control of tandem *lpp* and *lac*UV5 promoters	(1)
pBRα:P7	Amp	Encodes α-NTD (residues 1-248) of the α-subunit of *Ec* RNAp fused via three alanine residues to Xp10 P7 under control of tandem *lpp* and *lac*UV5 promoters	this work
pBRα:P7 F50A	Amp	pBRα:P7 where P7 harbour F50A substitution, deficient in interaction with β'-NTD	this work
pBRα:P7 V51A	Amp	pBRα:P7 where P7 harbour V51A substitution, deficient in interaction with β'-NTD	this work
pBRα:P7 L67A	Amp	pBRα:P7 where P7 harbour L67A substitution, deficient in interaction with β'-NTD	this work
pBRα:P7 R60E	Amp	pBRα:P7 where P7 harbour R60E substitution, deficient in interaction with β flap domain	this work
pBRα:P7Δ(67-74)	Amp	Encodes α-NTD (residues 1-248) of the α-subunit of *Ec* RNAp fused via three alanine residues to truncated variant of Xp10 P7 (residues 1-66) under control of tandem *lpp* and *lac*UV5 promoters	this work
pACλI	Cm	Encodes residues 1-236 of λCI protein of bacteriophage λ under the control of *lac*UV5 promoter.	(2)
pACλI-β'Xo (1–10)	Cm	Encodes residues 1-236 of λCI protein of bacteriophage λ fused via three alanine residues to residues 1–10 of the β'-subunit of *Xo* RNAp under the control of *lac*UV5 promoter.	this work
pACλI-β'Xo (1–85)	Cm	Encodes residues 1-236 of λCI protein of bacteriophage λ fused via three alanine residues to residues 1–85 of the β'-subunit of *Xo* RNAp under the control of *lac*UV5 promoter	(3)
pACλI-β'Xo (1–95)	Cm	Encodes residues 1-236 of λCI protein of bacteriophage λ fused via three alanine residues to residues 1–95 of the β'-subunit of *Xo* RNAp under the control of *lac*UV5 promoter	this work
pACλI-β'Xo (11–95)	Cm	Encodes residues 1-236 of λCI protein of bacteriophage λ fused via three alanine residues to residues 1–95 of the β'-subunit of *Xo* RNAp under the control of *lac*UV5 promoter	this work
pACλI-β'Xo (63–95)	Cm	Encodes residues 1-236 of λCI protein of bacteriophage λ fused via three alanine residues to residues 63–95 of the β'-subunit of *Xo* RNAp under the control of *lac*UV5 promoter	this work
pACλI-σ70 region 4	Cm	Encodes residues 1-236 of λCI protein of bacteriophage λ fused via three alanine residues to residues 528-613 of σ^{70}-subunit of *Ec* RNAp under the control of *lac*UV5 promoter.	(4)
pACλI-β flap (831–1057)	Cm	Encodes residues 1-236 of λCI protein of bacteriophage λ fused via three alanine residues to residues 831–1057 of the β-subunit of *Ec* RNAp under the control of *lac*UV5 promoter.	(5)
pACλI-β flap (831–1057) ΔFTH	Cm	pACλI flap (831–1057) where the β flap-tip helix (residues 900-909 of β) is deleted	(5)
pACλI-β flap (831–1057) L901A	Cm	pACλI flap (831–1057) encoding the L901A substitution in the β moiety of the fusion	(5)
pACλI-β flap (831–1057) L905A	Cm	pACλI flap (831–1057) encoding the L905A substitution in the β moiety of the fusion	(5)
pRL663	Amp	Allow expression *Ec* C-6his-β' from IPTG inducible promoter	(2)
pRL663:NLFN	Amp	Allow expression NLFN- *Ec* C-6his-β' from IPTG inducible promoter	(3)
pET33b	Km	Expression vector with T7 promoter, allowing phosphorylation of expressing product by Heart Muscle Kinase	Novagene
pET33:rpoD	Km	Overexpression of *Ec* N-6his-HMK-RpoD	this work
pET33:P7	Km	Overexpression of N-6His-HMK-P7	this work
pET33:P7(R60E)	Km	Overexpression of N-6His-HMK-P7 (R60E)	this work
pET46	Amp	Expression vector with T7 promoter and EK/LIC sites	Novagene
pET46:P7	Amp	Overexpression of N-6His-P7	this work

1. Dove, S.L., Joung, J.K. and Hochschild, A. (1997) Activation of prokaryotic transcription through arbitrary protein-protein contacts. *Nature*, **386**, 627-630.
2. Arthur, T.M. and Burgess, R.R. (1998) Localization of a sigma70 binding site on the N terminus of the *Escherichia coli* RNA polymerase beta' subunit. *The Journal of Biological Chemistry*, **273**, 31381-31387.
3. Yuzenkova, Y., Zenkin, N. and Severinov, K. (2008) Mapping of RNA polymerase residues that interact with bacteriophage Xp10 transcription antitermination factor p7. *Journal of Molecular Biology*, **375**, 29-35.
4. Yuan, A.H., Gregory, B.D., Sharp, J.S., McCleary, K.D., Dove, S.L. and Hochschild, A. (2008) Rsd family proteins make simultaneous interactions with regions 2 and 4 of the primary sigma factor. *Molecular Microbiology*, **70**, 1136-1151.
5. Deighan, P., Diez, C.M., Leibman, M., Hochschild, A. and Nickels, B.E. (2008) The bacteriophage lambda Q antiterminator protein contacts the beta-flap domain of RNA polymerase. *Proceedings of the National Academy of Sciences of the United States of America*, **105**, 15305-15310.

Table S3. NMR and refinement statistics

	P7	P7-β' NTD complex
**NMR Distance and Dihedral Constraints**		
Distance constraints		
Total NOE	941	1440
Intraresidue	385	429
Interresidue	536	888
Sequential (|*i*-*j*|)=1)	190	273
Medium range (|*i*-*j*|)<4)	30	82
Long range (|*i*-*j*|)>5)	231	369
Intermolecule	Nil	123
Total Dihedral angle Restraints	108	114
φ	54	57
ψ	54	57
**Structural Statistics**		
Violations (mean and SD)		
Distance constraints (Å)	0.017 ± 0.004	0.016 ± 0.004
Dihedral angle constraints (°)	0.33 ± 0.042	0.34 ± 0.043
Maximum dihedral angle violation (°)	0.22	0.24
Maximum distance constraint violation (Å)	0.252	0.180
Deviations from idealized geometry		
Bond length (Å)	0.003 ± 0.000	0.003 ± 0.000
Bond angle (°)	0.49 ± 0.007	0.55 ± 0.007
Impropers (°)	0.84 ± 0.01	1.41 ± 0.01
Average Pairwise rmsd[α](Å)		
Heavy	0.628	0.675
Backbone	0.260	0.220
